# Research papers by radiographers and radiation therapists: An Australian perspective

**DOI:** 10.1002/jmrs.32

**Published:** 2013-12-02

**Authors:** Michal Schneider

**Affiliations:** Department of Medical Imaging and Radiation Science, Monash University 3800 Clayton, VIC, Australia Tel: +61 3 99051348

Re: Snaith B. An evaluation of author productivity in international radiography journals 2004–2011. *J Med Radiat Sci* 2013; **60**(3): 93–9.

I read with interest this article which aims to evaluate productivity of radiographers and radiation therapists with regard to publications between 2004 and 2011 in four selected discipline-specific journals.

The authors have provided a comprehensive analysis of author productivity and ranked the authors according to number of articles published in these journals within the given time period. While this approach may appear to showcase the productivity by some authors, overall it does, in my opinion, not accurately reflect publication and research activity by radiographers and radiation therapists, at least not in Australia. The predominant reason that underlies this belief is the fact that only three of the four journals selected for evaluation are indexed in any electronic search engines. (The author has stated that the journal *Radiography* is not listed on the ‘Scopus’ database. However, it does appear in Scopus, at least in 2013.)

Most radiographers and radiation therapists select alternative journals and aim to publish in high-impact journals (relative to their discipline) and journals that can reach the most relevant readership efficiently. Radiography-specific journals commonly do not fulfil these criteria.

Given the publication records of our radiography and radiation therapy collaborators, as well as our current and former research students, it is clear that the article by Snaith only delivers a very small snapshot within a highly selected group of journals that have no impact factors and are not disseminated widely. I acknowledge the difficulty in evaluating this topic; however, I am afraid that the conclusions of this article do not accurately convey the research contributions of an increasing number of research-active professionals in this field.

In order to achieve due recognition and promote and disseminate the work of radiographers and radiation therapists, we need to ensure that these discipline-specific journals are indexed in electronic databases, have an impact factor, and are appropriately peer reviewed. Once we have achieved this, the quality of published work will improve, citations will increase and the h-indices will rise. All these will ultimately improve the profile of radiography and radiation therapy research.

